# Associations of abdominal obesity-related dietary patterns with prediabetes and type 2 diabetes: exploring the mediating effects of body composition and altitude in Tibetan adults

**DOI:** 10.1017/S1368980025100724

**Published:** 2025-07-18

**Authors:** Bin Zhang, Ruihua Xu, Zumin Shi, Tingting Wang, Wenxiu Jian, Haijing Wang, Ruijie Xu, Lei Zhao, Youfa Wang, Wen Peng

**Affiliations:** 1 School of Mathematics and Statistics, Qinghai Minzu University, Xining 810007, People’s Republic of China; 2 Nutrition and Health Promotion Center, Department of Public Health, Medical College, Qinghai University, Xining 810008, People’s Republic of China; 3 Qinghai Provincial Center for Disease Control and Prevention, Xining 810021, People’s Republic of China; 4 Department of Nutrition Sciences, College of Health Sciences, QU Health, Qatar University, Doha 2713, Qatar; 5 Global Health Institute, School of Public Health, Xi’an Jiaotong University, Xi’an 710061, People’s Republic of China; 6 Qinghai Provincial Key Laboratory of Prevention and Control of Glucolipid Metabolic Diseases with Traditional Chinese Medicine, Xining, People’s Republic of China

**Keywords:** Tibetan, Dietary patterns, Type 2 diabetes, Prediabetes, Reduced rank regression

## Abstract

**Objectives:**

To investigate the association of dietary patterns (DP) with prediabetes and type 2 diabetes (T2D) among Tibetan adults, first to identify DP associated with abdominal obesity and examine their relationships with prediabetes and T2D. Additionally, the study aims to investigate the mediating effects of body fat distribution and altitude on the associations between these DP and the prevalence of prediabetes and T2D.

**Design:**

An open cohort among Tibetans.

**Setting:**

Community-based.

**Participants:**

The survey recruited 1003 participants registered for health check-ups from November to December 2018 and 1611 participants from December 2021 to May 2022. During the baseline and follow-up data collection, 1818 individuals participated in at least one of the two surveys, with 515 of them participating in both.

**Results:**

Two DP were identified by reduced rank regression. DP1 had high consumption of beef and mutton, non-caloric drink and offal and low intake in tubers and roots, salty snacks, onion and spring onion, fresh fruits, desserts and nuts and seeds; DP2 had high intake of whole grains, Tibetan cheese, light-coloured vegetables and pork and low intake of sugar-sweetened beverages, whole-fat dairy products and poultry. Individuals in the highest tertile of DP1 showed higher risks of prediabetes (OR 95 % CI) 1·35 (1·05, 1·73) and T2D 1·36 (1·05, 1·76). The highest tertile of DP2 exhibited an elevated risk of T2D 1·63 (1·11, 2·40) in full adjustment.

**Conclusion:**

Abdominal adiposity-related DP are positively associated with T2D. Promoting healthy eating should be considered to prevent T2D among Tibetan adults.

Type 2 diabetes (T2D) poses a major worldwide health issue, characterised by its widespread occurrence and notable effects on illness and death rates. With the ongoing increase in its occurrence, T2D is becoming a significant public health issue, imposing extensive medical and financial costs on global healthcare systems. Forecasted trends indicate an astonishing surge in T2D instances, escalating from 463 million in 2019 to a projected 700 million by 2045^(1)^. As the number of T2D cases alarmingly increases, prediabetes being an early indicator highlights the critical need for active intervention tactics to reduce the looming impact of blatant diabetes. Particularly in China, the increase in diabetes cases from below 1 % in the 1980s to 11·2 % in 2015, coupled with a 38·1 % prediabetes percentage in 2018^(2)^, underscores the need for focused attention on both prediabetes and diabetes among particular groups. Tibetan adults are especially worrying due to noted differences in understanding, managing and controlling diabetes in their group^(3)^. The significant rise and imbalance in prediabetes and diabetes rates among Tibetan adults necessitate enhanced focus on personal and community health care.

The aetiology of T2D is complex, intricately linked with a range of reversible elements like exercise, dietary habits and lifestyle and irreversible aspects such as age, ethnicity and genetic predispositions^(4–6)^. Among the alterable risk elements linked to T2D, unhealthy eating patterns are notably prominent. Dietary habits in the West, marked by a substantial intake of red and processed meats, refined grains, fats, sweets and sodas, are associated with an increased risk of T2D^(7,8)^. On the other hand, following a Mediterranean diet that promotes health, abundant in plant-based foods, olive oil, seafood and moderately consuming nuts and dairy products, is linked to a lower occurrence of CVD and diabetes^(9,10)^. Nonetheless, the suitability of these established dietary habits for varied subgroups faces obstacles due to elements like food availability, cultural eating habits and regional variations. The pastoral regions of the Tibetan Plateau, characterised by high altitudes and sparse air, harbour unique lifestyle and dietary characteristics among local Tibetan residents, mainly engaged in animal husbandry and subsisting on staples such as tsamba, a traditional high calories and high-fat Tibetan food (roasted Tibetan barley, mixed with butter and sugar), Tibetan cheese, ghee tea/milk tea and dairy products^(11,12)^. In recent years, the social and economic face of Tibetan herdsmen has undergone major shifts, propelled by policies to promote ecological migration, nomadic settlement construction and poverty alleviation initiatives. Consequently, an increasing number of Tibetan herders are transitioning from higher elevation pastoral areas to lower elevation urban centres, resulting in profound changes in their living environments, livelihoods and eating habits^(13,14)^. Notably, these shifts have been accompanied by a shift towards a Westernised dietary pattern characterised by increased consumption of high-fat and ultra-processed foods^(15)^.

These environmental and lifestyle disparities foster unique adaptations among Tibetan populations, influencing metabolic processes, body composition and other physiological parameters crucial for adaptation to hypoxic conditions^(16,17)^. Particularly, Tibetans exhibit elevated basal metabolic rates, enhanced respiratory functions and thermoregulatory mechanisms to compensate for the challenges posed by high altitudes^(18,19)^. Genetic signals implicated in metabolic adaptations further underscore the complex interplay between genetic predispositions and environmental influences, influencing body fat composition and metabolic outcomes^(20,21)^. The distribution of body fat, particularly the ratio between android (central) and gynoid (peripheral) fat masses, holds implications for metabolic health and T2D risk^(22)^, with increased visceral fat mass being associated with heightened insulin resistance^(23)^.

Given the sophisticated interplay between dietary patterns (DP), body composition and metabolic health, elucidating these relationships in high-altitude settings is imperative. Employing reduced rank regression (RRR), a statistical technique adept for identifying dietary patterns^(24)^ associated with disease outcomes through their correlations with body composition variables, offers a comprehensive understanding of the dietary determinants of T2D and prediabetes. In this study, we employed RRR to explore the associations between abdominal obesity-related DP and prediabetes/T2D outcomes of migrating Tibetan adults. Additionally, the study also aims to investigate the mediating effects of body fat distribution and altitude on the associations between these DP and prediabetes/T2D. By shedding light on these complex interactions, our study aims to inform targeted interventions tailored to the unique dietary^(14)^ and metabolic profiles of Tibetan populations, thereby mitigating the burgeoning burden of T2D and prediabetes within these communities.

## Material and methods

### The participants and study design

In this community-based, open cohort study, which recruited 1818 subjects aged 18–91 years from the general population between 2018 and 2022. Participants originate from two communities in Golmud City, situated at an altitude of 2800 metres, gradually transitioning or semi-settling in urban or suburban areas from high-altitude pastoral regions (> 4000 metres) since 2007^(12)^. In this study, 1818 participants completed at least one wave of the two surveys that included baseline and follow-up data collection, and 515 of the participants completed two waves of the surveys (see online supplementary material, Supplemental Figure S1). The data contained questionnaire surveys, anthropometric measurements and biochemical tests. In the present study, exclusion criteria encompassed individuals under 18 years old (*n* 5), non-Tibetan participants (*n* 31), incomplete dietary survey responses (*n* 105) and those lacking outcome variables (*n* 140) (see online supplementary material, Supplemental Figure S1). Ethics approval was obtained from the Ethics Committee of Qinghai University Medical School, with written informed consent secured from all participants.

### Dietary assessment and dietary patterns

Face-to-face interviews utilising an updated FFQ facilitated the collection of dietary intake data, assessing self-reported food consumption over the preceding year. The FFQ, developed based on nutritional similarities and local dietary customs, aligns with prior research by Peng *et al.* ^(14)^. Food consumption frequencies were standardised to a monthly basis. A total of fifty food items were classified into twenty-six food groups.

### Outcome ascertainment of prediabetes and type 2 diabetes

Blood samples obtained from participants underwent analysis for fasting blood glucose and glycated Hb, performed by certified laboratories in Golmud Second People’s Hospital. According to the standard of WS/T 428–2013 (China), overweight and obesity are defined by BMI – overweight if 24 kg/m^2^ ≤ BMI < 28 kg/m^2^ and obesity if BMI ≥ 28 kg/m^2(25,26)^. T2D and prediabetes were defined in alignment with the 2020 Chinese Guidelines for the Prevention and Treatment of Type 2 Diabetes (T2D was defined as fasting glucose ≥ 7·0 mmol/l or HbA1c ≥ 6·5 % or self-reported diabetes; prediabetes was defined as 6·1 mmol/l ≤ fasting glucose < 7·0 mmol/l or 5·7 % ≤ HbA1c < 6·5 %)^(27)^.

### Body composition measurement

Body fat compositions, including visceral fat mass, abdominal fat mass, android fat mass and gynoid fat mass, were assessed via DXA (Hologic Horizon W) in fan beam mode with Hologic Apex software (version 4.0). Certified operators trained by the International Society for Clinical Densitometry conducted measurements, with indirect body composition components derived subsequently in the analysis.

### Covariate assessment

Structured questionnaires captured sociodemographic and lifestyle factors, including age, gender, marital status (unmarried/widowed/divorced/separated, married), educational level (no schooling, < 6 years of schooling, ≥ 6 years of schooling), household income (< 20 000, 20 000–100 000, > 100 000 CNY), smoking status (never smoking, former smoker, current smoker), alcohol consumption (never drinking, former drinker, current drinker), physical activity (light, moderate, heavy) and altitude (very high: participants with residence time more than 4 months at pasturing area, high altitude: the rest).

### Mediation analyses

Mediation analysis explored the association between DP scores (exposure), prediabetes/T2D (outcomes) and body composition^(28)^ and altitude (mediator)^(29)^. The mediation R package facilitated estimation of average direct effects, average causal mediation effects, total effects and proportion of mediated effects through bootstrap simulations.

### Statistical analyses

Participant characteristics were presented using appropriate descriptive statistics (mean (sd) for continuous data and as *n* (%) for categorical data. Student’s *t* test or ANOVA was employed for comparing means. The χ^2^ test was used for comparing the differences between categorical variables. RRR is a dietary analysis method that integrates prior knowledge with data-driven approaches. It identifies DP associated with diseases through intermediate response variables, which are typically mediators linked to disease risk. Compared with data-driven methods such as principal component analysis and cluster analysis, RRR has greater aetiological advantages. The DP derived from RRR are more likely to be connected with biological pathways related to disease aetiology^(30)^. Extensive research has shown that body fat distribution, particularly abdominal fat accumulation, is a risk factor for diabetes and prediabetes^(31,32)^. In this study, we selected intermediate response variables (visceral fat mass/abdominal fat mass, android fat mass/gynoid fat mass and waist-to-hip ratio (WHR)) related to adiposity indicators, assessing the severity of obesity especially abdominal obesity more precisely. Consequently, the DP identified are specifically related to abdominal obesity. From twenty-six food groups, DP were identified using RRR linked to three body composition variables (visceral fat mass/abdominal fat mass, android fat mass/gynoid fat mass and WHR, with factor loadings exceeding 0·20 considered significant contributors to a specific abdominal obesity-related DP. Factor scores categorised DP into tertiles, representing low (T1), medium (T2) and high score (T3) groups, respectively. Mixed-effects logistic regression models were employed to evaluate the relationship between DP tertiles and diseases, with adjustments made for relevant covariates across multiple models (Model 1 was adjusted for age and gender; Model 2 was further adjusted for marital status, education, insurance, household income, smoking, drinking and physical activity; Model 3 was additionally adjusted for altitude; Model 4 was similar to Model 3 but only included those with data at both baseline and follow-up). Statistical analyses were performed using Stata (version 17.0) and R software (version 4.2.3), with significance set at *P* < 0·05 (two-tailed).

## Results

### Dietary pattern related to prediabetes and type 2 diabetes

Over the study duration from 2018 to 2022, a total of 1818 participants were included, with 515 attending twice and 1303 observed once (see online supplementary material, Supplemental Figure S1). Participants had a mean age of 43·1 (sd 14·4) years, with men comprising 45·9 % and women 54·1 %. The majority exhibited low educational levels, hailed from rural areas, lacked insurance coverage and reported average incomes ranging between 20 000 and 100 000 CNY. They engaged in light physical activities, abstained from smoking and alcohol consumption (Table 1). The prevalence rates of prediabetes and T2D were 32·5 and 10·5 %, respectively.

**Table 1.** Demographic and lifestyle characteristics of participants by two dietary pattern (DP) scores in Tibetan communities (*n* 1818)

	All		DP1							DP2						
			T1		T2		T3		*P*	T1		T2		T3		*P*
	*n* 1818		*n*1 = 606		*n*2 = 606		*n*3 = 606			*n*1 = 606		*n*2 = 606		*n*3 = 606		
	*n*	%	*n*	%	*n*	%	*n*	%		*n*	%	*n*	%	*n*	%	
Survey year (%)									< 0·001							< 0·001
2018	935	51·4 %	350	57·8 %	296	48·8 %	289	47·7 %		397	65·5 %	318	52·5 %	220	36·3 %	
2022	883	48·6 %	256	42·2 %	310	51·2 %	317	52·3 %		209	34·5 %	288	47·5 %	386	63·7 %	
	Mean	sd	Mean	sd	Mean	sd	Mean	sd		Mean	sd	Mean	sd	Mean	sd	
Age (years)	43·1	14·4	40·3	14·2	43·8	14·5	45·3	14·0	< 0·001	38·8	13·4	44·7	14·6	45·8	14·1	< 0·001
	*n*	%	*n*	%	*n*	%	*n*	%		*n*	%	*n*	%	*n*	%	
Age (years, %)									< 0·001							< 0·001
18∼	979	53·9 %	382	63·0 %	313	51·7 %	284	46·9 %		398	65·7 %	303	50·0 %	278	45·9 %	
45∼	598	32·9 %	164	27·1 %	202	33·3 %	232	38·3 %		166	27·4 %	204	33·7 %	228	37·6 %	
60∼	241	13·3 %	60	9·9 %	91	15·0 %	90	14·9 %		42	6·9 %	99	16·3 %	100	16·5 %	
Sex									0·002							0·130
Men	835	45·9 %	244	40·3 %	290	47·9 %	301	49·7 %		296	48·8 %	278	45·9 %	261	43·1 %	
Women	983	54·1 %	362	59·7 %	316	52·1 %	305	50·3 %		310	51·2 %	328	54·1 %	345	56·9 %	
Marital (%)									0·600							0·120
Unmarried/widowed/divorced/separated	251	13·8 %	85	14·0 %	89	14·7 %	77	12·7 %		98	16·2 %	75	12·4 %	78	12·9 %	
Married	1566	86·2 %	521	86·0 %	517	85·3 %	528	87·3 %		508	83·8 %	530	87·6 %	528	87·1 %	
Education (%)									< 0·001							< 0·001
No schooling	1321	72·7 %	404	66·7 %	442	72·9 %	475	78·4 %		392	64·7 %	456	75·2 %	473	78·1 %	
< 6 years of schooling	158	8·7 %	60	9·9 %	46	7·6 %	52	8·6 %		58	9·6 %	55	9·1 %	45	7·4 %	
≥ 6 years of schooling	339	18·6 %	142	23·4 %	118	19·5 %	79	13·0 %		156	25·7 %	95	15·7 %	88	14·5 %	
Insurance (%)									0·012							< 0·001
Rural/No insurance	1095	60·2 %	337	55·6 %	386	63·7 %	372	61·4 %		268	44·2 %	396	65·3 %	431	71·1 %	
Urban insurance	723	39·8 %	269	44·4 %	220	36·3 %	234	38·6 %		338	55·8 %	210	34·7 %	175	28·9 %	
Household income (%)									0·084							< 0·001
< 2	421	23·9 %	156	26·6 %	138	23·5 %	127	21·5 %		119	20·2 %	168	28·9 %	134	22·6 %	
2∼10	1124	63·7 %	349	59·5 %	386	65·8 %	389	65·8 %		360	61·1 %	350	60·1 %	414	69·7 %	
10∼	220	12·5 %	82	14·0 %	63	10·7 %	75	12·7 %		110	18·7 %	64	11·0 %	46	7·7 %	
Smoking (%)									0·067							< 0·001
Never	1432	78·8 %	470	77·6 %	471	77·9 %	491	81·0 %		430	71·0 %	493	81·5 %	509	84·0 %	
Former smoker	110	6·1 %	31	5·1 %	48	7·9 %	31	5·1 %		43	7·1 %	34	5·6 %	33	5·4 %	
Current smoker	275	15·1 %	105	17·3 %	86	14·2 %	84	13·9 %		133	21·9 %	78	12·9 %	64	10·6 %	
Alcohol drinking (%)									0·550							0·015
Never	1521	83·7 %	503	83·0 %	502	82·8 %	516	85·1 %		485	80·0 %	520	85·8 %	516	85·1 %	
Abstinence	124	6·8 %	47	7·8 %	44	7·3 %	33	5·4 %		43	7·1 %	40	6·6 %	41	6·8 %	
Current alcohol drinker	173	9·5 %	56	9·2 %	60	9·9 %	57	9·4 %		78	12·9 %	46	7·6 %	49	8·1 %	
Physical activity (%)									0·630							0·230
Light	1118	61·6 %	383	63·2 %	360	59·6 %	375	62·0 %		394	65·1 %	356	58·8 %	368	60·8 %	
Moderate	460	25·3 %	143	23·6 %	160	26·5 %	157	26·0 %		140	23·1 %	161	26·6 %	159	26·3 %	
Heavy	237	13·1 %	80	13·2 %	84	13·9 %	73	12·1 %		71	11·7 %	88	14·5 %	78	12·9 %	
Living altitude^†^ (%)									0·064							0·076
High altitude	1075	72·4 %	377	75·2 %	367	73·1 %	331	68·7 %		290	68·2 %	371	73·9 %	414	74·2 %	
Very high altitude	410	27·6 %	124	24·8 %	135	26·9 %	151	31·3 %		135	31·8 %	131	26·1 %	144	25·8 %	
	Mean	sd	Mean	sd	Mean	sd	Mean	sd		Mean	sd	Mean	sd	Mean	sd	
BMI (kg/m^2^)	25·9	4·9	25·4	5·0	26·0	4·9	26·2	4·9	0·016	25·4	4·7	26·0	5·0	26·2	4·9	0·005
Waist (cm)	87·3	12·5	85·4	12·6	88·2	12·4	88·4	12·4	< 0·001	85·7	12·5	87·5	12·0	88·7	12·9	< 0·001
Hip (cm)	97·4	9·0	96·5	9·3	97·9	8·8	97·8	8·9	0·019	96·3	9·1	97·4	8·9	98·5	9·0	< 0·001
Waist-to-hip ratio)	1·0	0·1	0·9	0·1	1·0	0·1	1·0	0·1	< 0·001	0·9	0·1	1·0	0·1	1·0	0·1	0·049
	*n*	%	*n*	%	*n*	%	*n*	%		*n*	%	*n*	%	*n*	%	
Overweight (%)									< 0·001							0·190
< 24	714	57·6 %	273	64·8 %	225	54·2 %	216	53·5 %		264	60·4 %	236	57·8 %	214	54·2 %	
24∼28	526	42·4 %	148	35·2 %	190	45·8 %	188	46·5 %		173	39·6 %	172	42·2 %	181	45·8 %	
Obesity (%)									0·600							0·035
< 28	1240	68·5 %	421	69·8 %	415	68·6 %	404	67·1 %		437	72·4 %	408	67·5 %	395	65·6 %	
28∼	570	31·5 %	182	30·2 %	190	31·4 %	198	32·9 %		167	27·6 %	196	32·5 %	207	34·4 %	
Prediabetes (%)									< 0·001							< 0·001
No	1137	67·5 %	416	73·0 %	377	67·2 %	344	62·2 %		439	76·1 %	360	64·7 %	338	61·3 %	
Yes	547	32·5 %	154	27·0 %	184	32·8 %	209	37·8 %		138	23·9 %	196	35·3 %	213	38·7 %	
Diabetes (%)									0·039							< 0·001
No	1137	89·5 %	416	92·0 %	377	89·3 %	344	86·6 %		439	93·8 %	360	87·8 %	338	86·0 %	
Yes	134	10·5 %	36	8·0 %	45	10·7 %	53	13·4 %		29	6·2 %	50	12·2 %	55	14·0 %	

* Data are presented as the mean (sd) for continuous measures and as *n* (%) for categorical measures.

† Living altitude was defined as the living time of pasturing area. High altitude: less than 4 months. Very high altitude: more than 4 months.

**Figure 1.**
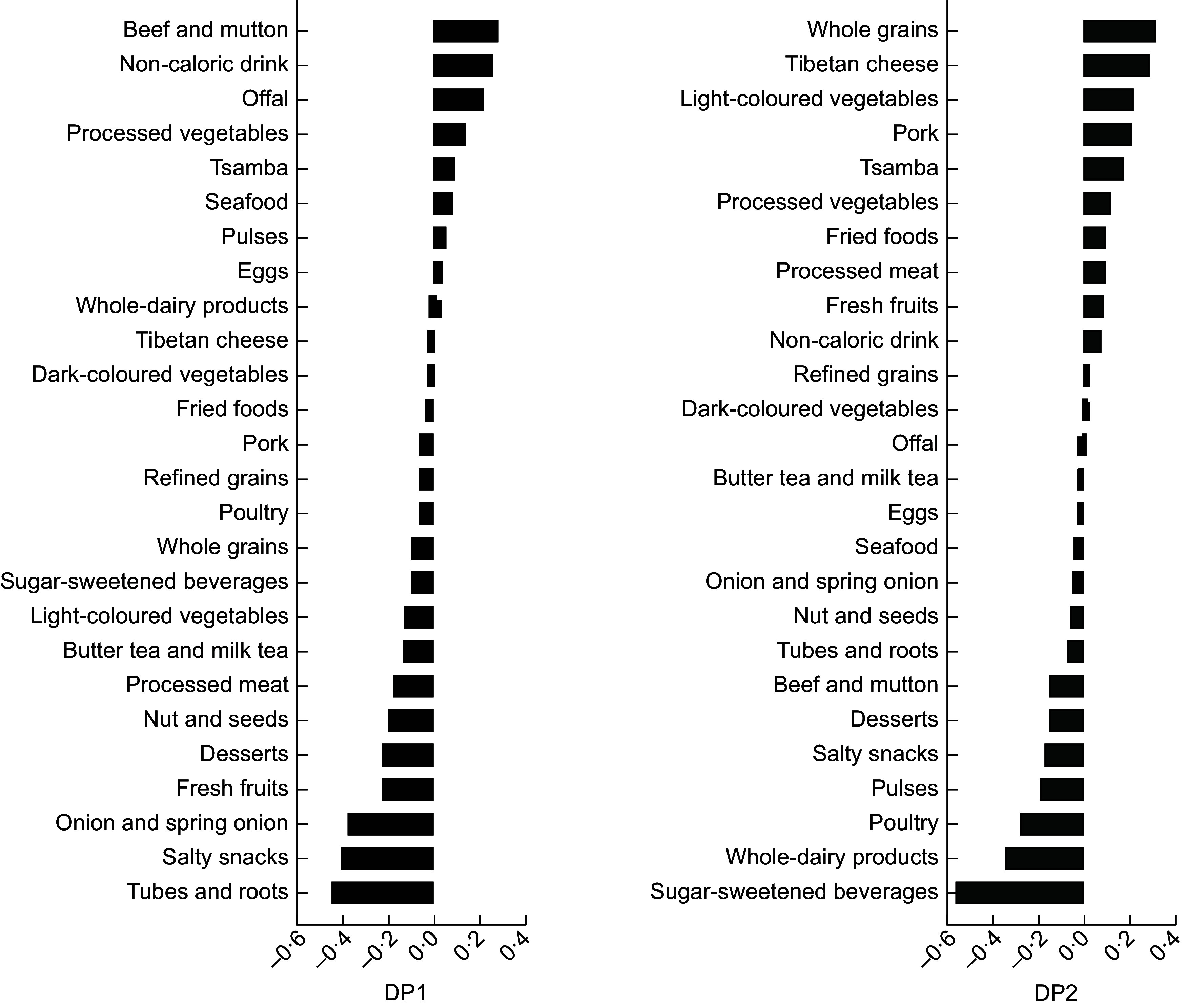
Tornado chart of factor loading of dietary patterns derived from a reduced rank regression from participants (*n* 827).

DP1 was characterised by elevated consumption of red meat, offal and non-caloric drinks, coupled with reduced intake of tubers and roots, salty snacks, onions, spring onions, fresh fruits, desserts, nuts and seeds. Conversely, DP2 featured high loadings of whole grains, Tibetan cheese, light-coloured vegetables and pork, along with decreased consumption of sugar-sweetened beverages, whole-fat dairy products and poultry (Figure 1). These DP represent two distinct Tibetan DP, accounting for 3·87 and 4·25 % of the total intermediary variance, respectively, and explaining 4·38 and 7·89 % of the variance in total food intake. DP1 and DP2 accounted for variances in log-transformed intermediary variables, including visceral fat mass/abdominal fat mass, android fat mass/gynoid fat mass and WHR, ranging from 1·52 to 5·28 % (Table 1).

### Characteristics of the participants among two different dietary patterns

Table 1 demonstrates the demographic and lifestyle characteristics of 1818 participants based on the tertiles of two DP scores. Participants with high DP1 scores (T3) exhibited an older age (45·3 (sd 14·0) years) and elevated prevalence rates of overweight, obesity, prediabetes and diabetes (46·5 %, 32·9 %, 37·8 %, 13·4 %, respectively). Conversely, individuals with high DP2 scores (T3) predominantly resided at high altitudes (74·2 %) and presented with higher BMI (26·2 (sd 4·9)), waist circumference (88·7 (sd 12·9) cm), hip circumference (98·5 (sd 9·0) cm) and WHR (1·0 (sd 0·1)). Notably, higher DP scores correlated with increased likelihood of prediabetes and diabetes.

### Associations between tertiles of dietary pattern scores with prediabetes and type 2 diabetes

Mixed-effects logistic regression models revealed associations between tertiles of DP scores and prediabetes/T2D (Table 2). In Model 3, adjusting for sociodemographic factors, lifestyle factors and altitude, higher DP1 and DP2 scores were associated with increased risk of prediabetes (for DP1with the OR (95 % CI) for the T1, T2 and T3: were 1·00, 0·98 (0·75, 1·27), 1·35 (1·05, 1·73) with a *P* trend 0·027 and 1·00, 1·24 (0·95, 1·61), 1·36 (1·05, 1·76) with a *P* trend 0·020 for DP2), but not consistently with T2D. However, in Model 2, DP2 scores showed a significant positive correlation with T2D (*P* trend = 0·013).

**Table 2.** Associations between tertiles of two dietary pattern (DP) scores with diabetes, prediabetes among Tibetans in China (*n* 2333 person-time)

DP1							DP2					
	T1	T2		T3		*P* trend	T1	T2		T3		*P* trend
		OR	95 % CI	OR	95 % CI			OR	95 % CI	OR	95 % CI	
Median	−0·31	0·01		0·25			−0·10	−0·01		0·06		
Prediabetes												
Crude	1	1·10	0·89, 1·38	1·56	1·26, 1·94	< 0·001	1	1·61	1·29, 2·00	1·85	1·49, 2·31	< 0·001
Model 1	1	0·96	0·76, 1·21	1·30	1·04, 1·63	0·028	1	1·34	1·06, 1·69	1·50	1·19, 1·89	< 0·001
Model 2	1	0·92	0·72, 1·17	1·17	0·92, 1·48	0·231	1	1·38	1·08, 1·76	1·53	1·20, 1·95	< 0·001
Model 3	1	0·98	0·75, 1·27	1·35	1·05, 1·73	0·027	1	1·24	0·95, 1·61	1·36	1·05, 1·76	0·020
Model 4	1	0·88	0·58, 1·34	1·34	0·92, 1·96	0·158	1	1·12	0·75, 1·69	1·28	0·87, 1·87	0·215
Diabetes												
Crude	1	1·33	0·92, 1·91	1·40	0·98, 2·00	0·050	1	2·12	1·49, 3·02	2·08	1·48, 2·92	< 0·001
Model 1	1	1·13	0·79, 1·61	1·28	0·90, 1·83	0·179	1	1·58	1·07, 2·32	1·52	1·05, 2·19	0·023
Model 2	1	1·05	0·73, 1·50	1·06	0·74, 1·53	0·737	1	1·71	1·13, 2·58	1·63	1·11, 2·40	0·013
Model 3	1	1·06	0·70, 1·61	1·20	0·79, 1·83	0·392	1	1·52	0·95, 2·41	1·52	0·97, 2·38	0·072
Model 4	1	0·98	0·50, 1·94	0·89	0·49, 1·61	0·707	1	1·53	0·78, 2·99	1·61	0·88, 2·94	0·127

* Crude was an unadjusted model. Model 1 was adjusted for age and sex; Model 2 was further adjusted for marital status, education, insurance status, household income, smoking status, alcohol consumption and physical activity; Model 3 was further adjusted for living altitude; Model 4 was the same as Model 3 but only included those who had both baseline and follow-up data. DP scores were equally divided into thirds. T means tertiles (T1–T3). Tibetan adults who attended at least one wave were included in 2018 and 2022 (*n* 2333 person-time).

**Figure 2.**
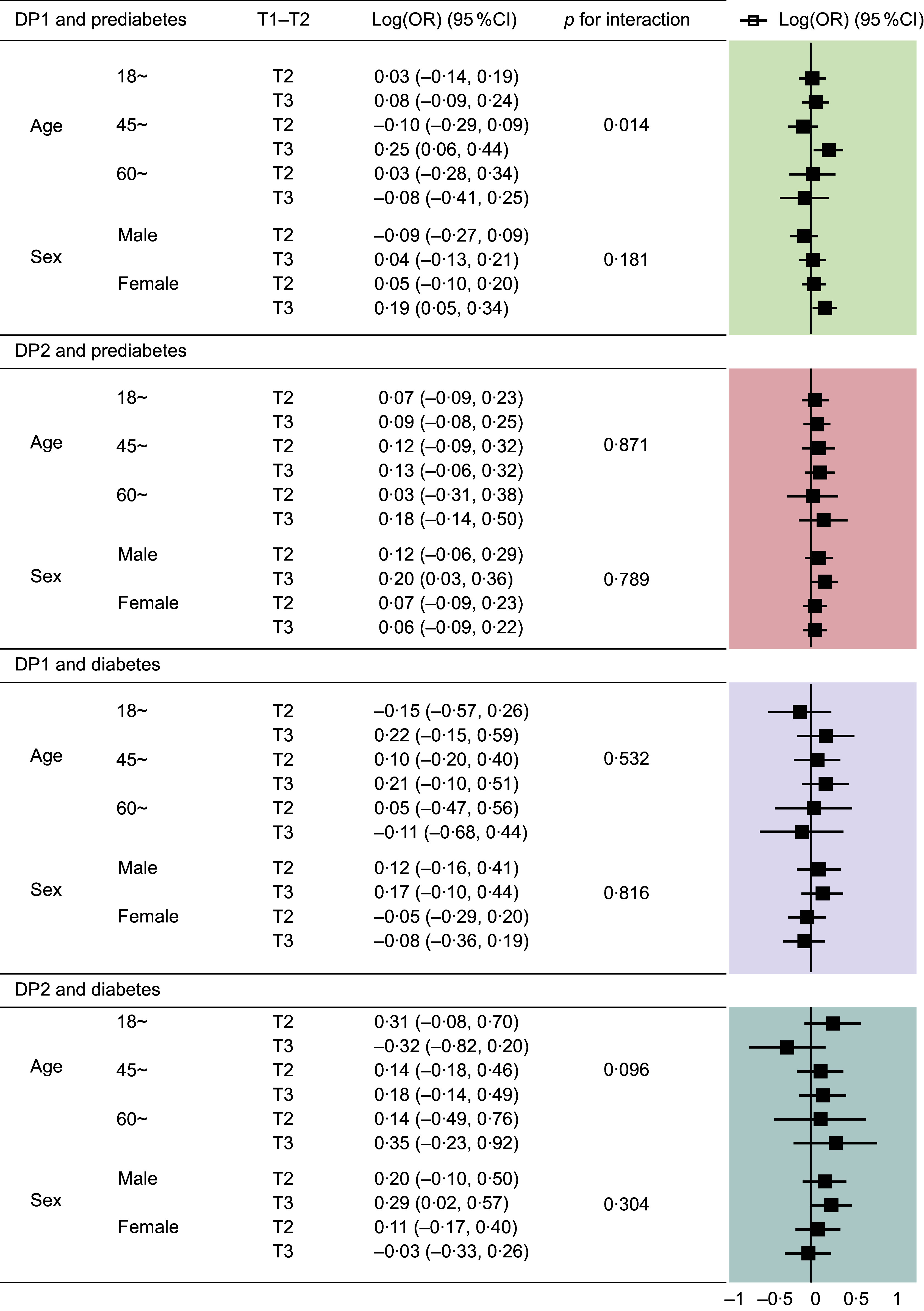
OR plot and 95 % CI of diabetes and prediabetes incidence, stratified by sex and age, according to the tertiles of dietary pattern1 and dietary pattern2 from subgroup analyses (*n* 2333). Reference group T1 is not shown in the figure.

Subgroup analysis highlighted significant interactions between DP and age in prediabetes prevalence (*P*_interaction_ < 0·05), with individuals aged 45–60 in high DP1 tertiles exhibiting the highest likelihood of prediabetes (Figure 2).

### Body fat distribution demonstrates mediation effects on dietary patterns

Mediation analysis elucidated the role of adipose tissues in the relationship between DP and outcomes (Table 3). Visceral fat mass/abdominal fat mass and android fat mass/gynoid fat mass significantly mediated the associations between DP1 and prediabetes/T2D: visceral fat mass/abdominal fat mass and android fat mass/gynoid fat mass had significant mediating effects in the correlation of prediabetes and T2D; the indirect effects OR (95 % CI) and the proportion of the indirect effect and *P* value were 1·06 (1·03, 1·08), 47·5 %, *P* < 0·001, 1·06 (1·04, 0·59), 53·8 %, *P* < 0·001 for prediabetes; and the indirect effects OR (95 % CI) and the proportion of the indirect effect and *P* value were 1·15 (1·09, 1·21), 54·5 %, *P* < 0·001, 1·19 (1·12, 1·25), 70·5 %, *P* < 0·001 for T2D, respectively. Notably, android fat mass/gynoid fat mass exerted the most substantial mediating effects in the association with prediabetes and T2D. Similarly, WHR exhibited mediating effects in the association between DP2 and prediabetes/T2D: WHR’s indirect effects OR (95 % CI) and the proportion of the indirect effect and *P* value were 1·08 (1·02, 1·15), 52·8 %, *P* < 0·01 for prediabetes, 1·18(1·05, 1·31), 27·9 %, *P* < 0·01 for diabetes respectively. The mediating effect of altitude on prediabetes and diabetes was conducted; however, it was low compared with these body composition indices above, which is shown in online supplementary material, Supplemental Table S1.

**Table 3.** Mediating effects of three obesity indicators on the association between DP1 and DP2 with prediabetes and diabetes

	Total effects		Direct effects		Indirect effects		Proportion of indirect effect
	OR	95 % CI	OR	95 % CI	OR	95 % CI	
DP1 and prediabetes							
Visceral fat mass/abdominal fat mass	1·12	1·02, 1·22	1·06	0·96, 1·17	1·06	1·03, 1·08	47·5 %
Waist-to-hip ratio (WHR)	1·12	1·02, 1·23	1·09	0·99, 1·20	1·03	1·00, 1·05	22·3 %
Android fat mass/gynoid fat mass	1·12	1·02, 1·22	1·05	0·95, 1·16	1·06	1·04, 1·09	53·8 %
DP2 and prediabetes							
Visceral fat mass/abdominal fat mass	1·15	0·83, 1·48	1·19	0·87, 1·51	0·97	0·90, 1·03	–
WHR	1·16	0·83, 1·48	1·07	0·76, 1·39	1·08	1·02, 1·15	52·8 %
Android fat mass/gynoid fat mass	1·15	0·82, 1·48	1·16	0·84, 1·48	0·99	0·93, 1·06	–
DP1 and diabetes							
Visceral fat mass/abdominal fat mass	1·29	1·12, 1·46	1·12	0·97, 1·28	1·15	1·09, 1·21	54·5 %
WHR	1·27	1·10, 1·44	1·17	1·00, 1·33	1·09	1·04, 1·13	35·0 %
Android fat mass/gynoid fat mass	1·28	1·11, 1·45	1·07	0·93, 1·21	1·19	1·12, 1·25	70·5 %
DP2 and diabetes							
Visceral fat mass/abdominal fat mass	1·77	1·29, 2·26	1·81	1·35, 2·27	0·98	0·88, 1·08	–
WHR	1·82	1·33, 2·31	1·54	1·07, 2·02	1·18	1·05, 1·31	27·9 %
Android fat mass/gynoid fat mass	1·80	1·28, 2·32	1·73	1·24, 2·21	1·04	0·92, 1·17	–

* ‘-’ means the indirect effects were NS.

## Discussion

A comprehensive understanding of the influence of DP on prediabetes and diabetes among Tibetan adults is critical for developing effective and suitable public health interventions tailored to this population. According to the National Surveillance Report, diabetes prevalence reached 11·9 % in 2018, with alarmingly low rates of awareness (38·0 %), treatment (34·1 %) and control (33·1 %)^(2)^. The high prevalence of prediabetes and diabetes poses a long-term challenge for Tibetans dwelling in high-altitude environments, potentially imposing significant burdens on local healthcare providers, both in terms of public health management and financial perspectives. Recognising the risk factors contributing to glucose tolerance issues is essential for devising targeted health promotion strategies aimed at reducing morbidity and mortality^(33)^ in this vulnerable population^(14)^. Moreover, the unique cultural traditions and lifestyles of Tibetan communities, coupled with limitations in food acquisition, contribute to distinct dietary habits that are not easily categorised into predefined DP such as Western or Mediterranean. Our study addresses this gap in the public health perspective, providing valuable insights into the relationship between dietary habits and metabolic health outcomes among Tibetan adults.

Our study identified two distinct DP related to abdominal adiposity associated with prediabetes/T2D. DP1 was characterised by high red meat, non-caloric drink, offal and low intake in tubers and roots, salty snacks, onion and spring onion, fresh fruits, desserts and nuts and seeds. Conversely, DP2 had high loadings of whole grains, Tibetan cheese, light-coloured vegetables and pork and low in sugar-sweetened beverages, whole-fat dairy products and poultry. Notably, high consumption of red meat emerged as a common key characteristic in both DP. This finding is consistent with previous research, such as a study examining the association between red meat intake and the risk of T2D among a large cohort^(34)^. The study reported a strong positive correlation between red meat consumption and the incidence of T2D, even after adjusting for BMI, suggesting an independent link between red meat intake and diabetes risk. The observed association between red meat intake and diabetes risk aligns with multiple biological mechanisms identified in previous research, including the effect of saturated fat, polyunsaturated fat, haem iron and processed meat components on insulin resistance and beta cell function. Saturated fat found in red meat has been shown to reduce beta cell function and insulin sensitivity, contributing to insulin resistance^(35)^. Additionally, the low content of polyunsaturated fat in red meat may further exacerbate insulin resistance, while haem iron, a component of red meat, increases oxidative stress and impairs beta cell function^(36–38)^. Processed red meats, in particular, contain high levels of nitrates and their byproducts, which promote endothelial dysfunction and insulin resistance^(39)^. While some observational studies have suggested that saturated and trans-fatty acids derived from whole-fat dairy products may be associated with a reduced risk of T2D^(40–42)^, the low consumption of whole-fat dairy products in DP2 aligns with these findings and is associated with an increased risk of T2D in our study. However, it is important to note that these associations are observational and do not imply causality.

As far as we are aware, this study adds to the scarce research exploring the link between eating habits and the likelihood of prediabetes and widespread diabetes in the Tibetan community. Significantly, our innovative method involved using RRR to clarify the links between eating habits and metabolic health results, integrating body composition metrics like WHR, visceral/abdominal fat mass and the fat mass ratio between android and gynoid areas. Adopting this all-encompassing strategy enabled a thorough evaluation of how DP affect metabolic well-being, considering the differences in body fat distribution among Tibetan adults, a factor not previously investigated in earlier research.

The results of our study highlight the critical role of central obesity, especially the buildup of visceral fat, in the emergence of prediabetes and diabetes in Tibetan people. Earlier studies have underscored the significance of visceral fat in metabolic wellness, linking it to insulin resistance and glucose intolerance. Our research sheds light on the impact of visceral fat as a possible risk factor for metabolic disorders in Tibetan communities, particularly in chronic high-altitude lifestyles where physiological changes to altitude might affect the distribution of body fat^(43)^. Additionally, the mediation study showed that WHR, an indicator of central obesity, significantly influenced the link between eating habits and prediabetes/T2D, underscoring the need to focus on central fat in preventative approaches. We conducted an analysis of the mediating effect of altitude due to the unique living conditions of Tibetan herders and its impact on DP and diabetes. However, the mediating effect of altitude on DP and prediabetes/T2D in this study was relatively weak, which may be attributed to the lower detection rate of diabetes in this region compared with other chronic non-communicable diseases such as hypertension, overweight and obesity^(44)^. Although it was relatively weak compared with body composition indices, it is worth noting that altitude may still play a role in metabolic health among Tibetan populations. Previous studies have suggested that high-altitude environments can influence metabolic adaptations, including changes in body composition and insulin sensitivity^(45,46)^. For instance, chronic exposure to high altitudes has been associated with altered fat distribution, which could indirectly affect diabetes risk^(47,48)^. However, the specific mechanisms through which altitude mediates dietary effects on metabolic outcomes remain unclear and warrant further investigation. Future studies with larger sample sizes and more detailed altitude-related data are needed to elucidate these relationships.

Strengths of our study include the comprehensive assessment of dietary intake using a tailored FFQ and the rigorous methodology employed in data collection and analysis. By considering traditional Tibetan foods and utilising standardised procedures for dietary assessment, we ensured the quality and accuracy of dietary data. Moreover, the use of RRR and mediation analysis allowed us to identify intermediary factors linking DP to metabolic outcomes, providing a deeper understanding of the underlying mechanisms.

Despite these strengths, our study has limitations that warrant consideration. First, the use of FFQ to assess dietary intake may introduce some inaccuracies, particularly regarding portion sizes^(14)^. Additionally, the open cohort study with relatively short follow-up time limits our ability to establish causal relationships between DP and prediabetes/T2D. Nevertheless, some participants with prediabetes are not aware of their glucose tolerance status upon the survey; it’s unlikely that reverse causation impacts our identified associations. This open cohort design incorporates a temporal sequence, offering stronger inferential power than a purely cross-sectional study. Furthermore, it utilises a larger sample size, enhancing the reliability of the results. Moreover, the consistency between Model 3 and Model 4 serves as a form of sensitivity analysis, demonstrating the robustness of our associations. This consistency suggests that our findings are less likely to be driven by reverse causation. Future research incorporating longitudinal data and larger sample sizes could be added to validate our findings and elucidate the underlying physiological mechanisms driving these associations. Nonetheless, our study contributes valuable insights into the dietary factors influencing metabolic health outcomes among Tibetan adults and underscores the importance of addressing central obesity in preventive interventions targeting prediabetes and diabetes within this population.

### Conclusions

Our study clarifies that the two identified DP were strongly associated with an increased risk of prediabetes and T2D in Tibetan adults. These associations were identified while considering body compositions such as visceral fat mass/abdominal fat mass, android fat mass/gynoid fat mass and WHR. This study highlights the necessity of tailoring dietary interventions to the unique cultural, dietary and logistical constraints of the Tibetan community, providing context-specific recommendations to improve dietary practices in a feasible and culturally sensitive manner.

## Supporting information

Zhang et al. supplementary material Zhang et al. supplementary material

## Data Availability

Some or all datasets generated during and/or analysed during the present study are not publicly available but are available from the corresponding author on reasonable request.

## Supplementary material

For supplementary material accompanying this paper, visit https://doi.org/10.1017/S1368980025100724.
