# Supplementary material for: Associations of abdominal obesity-related dietary patterns with prediabetes and type 2 diabetes: exploring the mediating effects of body composition and altitude in Tibetan adults
Source: Public Health Nutr. 2025 Jul 18;28(1):e131. doi: 10.1017/S1368980025100724 (PMC12465080; doi:10.1017/S1368980025100724)
Supplement: Zhang et al. supplementary material [file S1368980025100724sup001.docx]

**Figure S1.** The flowchart of participants.

Table S1. Mediating effects of altitude on the association between DP1 and DP2 with pre-diabetes and diabetes.

|  | **Total effects OR (95%CI)** | **Direct effects OR (95%CI)** | **Indirect effects OR (95%CI)** | **Proportion of indirect effect** |
| --- | --- | --- | --- | --- |
| **DP1&Prediabetes** |  |  |  |  |
| altitude | 1.08 (1.02, 1.15) | 1.08 (1.01, 1.15) | 1.01 (1.00, 1.01) | 5.80% |
| **DP2&Prediabetes** |  |  |  |  |
| altitude | 1.24 (1.03, 1.45) | 1.25 (1.04, 1.46) | 0.99 (0.98, 1.00) | ­­4.80% |
| **DP1&Diabetes** |  |  |  |  |
| altitude | 1.06 (1.00, 1.11) | 1.05 (1.00, 1.11) | 1.00 (1.00, 1.00) | 0.30% |
| **DP2&Diabetes** |  |  |  |  |
| altitude | 1.19 (1.02, 1.37) | 1.19 (1.01, 1.36) | 1.01 (0.99, 1.02) | 2.70% |
